# A computational case study of Günter Blobel’s idea of protein topogenesis and its influence

**DOI:** 10.1007/s40656-026-00732-7

**Published:** 2026-06-10

**Authors:** Shane T. Jinson, Jane Maienschein, Manfred Laubichler

**Affiliations:** 1 Department of Philosophy, University of California, Davis, USA; 2 The School of Life Sciences at Arizona State University, Tempe, AZ USA; 3 Marine Biological Laboratory, Woods Hole, MA USA; 4 The Santa Fe Institute, Santa Fe, NM USA; 5 School of Complex Adaptive Systems, Arizona State University, Tempe, AZ USA

**Keywords:** Quantitatively enhanced qualitative research, Detecting uncited influence, Computational linguistics, Cell biology, Protein

## Abstract

In this case study we present a novel way to assess scientific influence using computational linguistic tools. Here, we use such tools to identify semantic influence, or nuanced language patterns, with which to determine precedence of an idea and directionality of its spread. We aim to quantitatively test cell biologist and historian Karl Matlin’s claim that Günter Blobel’s ideas on protein topogenesis were more influential and had a larger impact on cell biology than the total number of citations of his 1980 paper “Intracellular Protein Topogenesis” would suggest. Examining collections of molecular cell biology journal articles from the 1980s through to the 2010s, we examine papers citing Blobel’s (Proc Natl Acad Sci 77(3):1496–1500, 1980. 10.1073/pnas.77.3.1496) paper, as well as relevant papers not citing it from the same time periods. We find changes in the language of these publications during the 1980s and early 1990s that can be traced back to Blobel’s introduction of the concept of *protein topogenesis*—or the spatial organizing of proteins that make up the cell—despite most cell biologists ignoring Blobel’s specific terms for sequences related to the phenomenon he describes, including the term “protein topogenesis” itself. We characterize language patterns from journal articles over different time periods for three different collections, each representing three communities of researchers: those citing Blobel’s paper, those citing different but related papers from around the same time, and a collection representative of the larger field relevant to each collection’s research topics. Finally, we demonstrate that the changes in language patterns of those discussing Blobel’s ideas in the 1980s and early 1990s are not limited to those directly citing his 1980 paper where the ideas were articulated. Specific terms related to the “protein topogenesis” concept first emerged within the network of researchers citing Blobel; the larger cell biology community then picked up these same language patterns and used them at similar rates five years after they were first detected in the smaller community of researchers *directly citing* his work. By deploying a suite of computational and quantitative methods representing auxiliary approaches to conducting history of science studies, we substantiate Matlin’s qualitative claim that the influence of Blobel’s “Intracellular Protein Topogenesis” far exceeds its citation count, and these ideas from Blobel somehow diffused into the larger community in a way that bypassed those directly citing these ideas.

## Background

Computational tools can be used to find novel insights using data (Damerow & Wintergrün, 2019), harnessing modern computing power to analyze large quantities of information from research publications (Laubichler et al., 2024) to find evidence of influence. In this paper, we use a traditionally-researched qualitative historical account in Karl Matlin’s *Crossing the Boundaries of Life* (Matlin, 2022) to quantitatively address the question: Did Günter Blobel’s *concept* of “protein topogenesis” outlined in his 1980 paper “Intracellular Protein Topogenesis” (Blobel, 1980), though not a term adopted by a significant number of molecular cell biologists, influence the larger field of molecular cell biology *beyond the small community of researchers who directly cited his 1980 paper?* Matlin, a former cell biologist and now a historian of cell biology, argues in a roundabout way that this is the case, and his assertions are based on his experience as a colleague of Blobel and others working in the field at the time. Our group used Matlin’s assertions and ideas put forward in his book as a jumping-off point to ask the question and hone criteria selection for this computational project, where we attempt to validate Matlin’s claim that Blobel *did* influence the larger field despite lacking a significant number of citations (Matlin, 2022).

There are many examples of how ideas spread beyond citations. Thomas Hunt Morgan’s “fly room” at Columbia University has been characterized as a hub of shared ideas and is an example of a small, centralized community from which influential ideas emerged and proliferated (Allen, 1978; Kohler, 1994). It was here that Calvin Bridges, Alfred Sturtevant, and other members of the Morgan lab would share and brainstorm ideas leading to the earliest genetic chromosomal maps and strains (as well as the earliest ideas of gene dynamics and concepts such as crossing over) by using *Drosophila melanogaster* as a model for genetic analysis (Lewis, 1998). Cold Spring Harbor was similarly a conduit of ideas in what Gunter Stent called the romantic phase of the informationist school in molecular biology (Stent, 1968). It served as a migratory meeting place in the summers, where the Phage course led by Max Delbrück and Salvador Luria became a sort of “watershed” leading to the development of ideas and tools used in molecular biology (Kohler, 1994; Olby, 1994).

Workers in different types of community structures—whether institutions (localized like the “fly room” at Columbia University), academic societies (distributed like the Society for Synthetic Biology), or meeting places (hybrid examples include Cold Spring Harbor or the Marine Biological Laboratory) are examples of spaces where ideas and influence are transferred—likely in ways unique to these different organizational structures. Traditional metrics of influence include counting the number of citations, or those that have so far cited a given paper since publication, of any given article, book, or other source of published information. The higher the number of citations, the more influential the paper. However, these traditional metrics of influence—such as merely counting the number of citations—offer limited insight regarding more subtle pathways of influence. In approaching our case study, we keep these organizational structures in mind (as we hone our approach) in order to design an experiment using multiple collections of entangled scientific literature from different research collections (e.g., an author-of-interest collection, a contemporary researcher’s collection, and even a collection representing the larger field not citing Blobel’s work directly). Our goal is to highlight the entanglements uniting these three collections within the context of the development of molecular cell biology and to detect evidence of uncited influence coming from one and spreading to another, potentially finding *directionality* for specific ideas between these sampled collections.

## Introduction to case

Günter Blobel won the Nobel Prize in 1999 for his signal hypothesis—his idea of how proteins are “tagged” for their eventual purpose in the cell (Anderson & Walter, 1999). From this idea, Blobel determined that parts of information sequences act as markers to match proteins for destinations during biological processes. However, Blobel made other contributions to the field of cell biology that, while adjacent to his work for which he won the Nobel Prize, were distinct in terms of their novelty and subsequent influence. In his chapter on “Topogenesis and Spatial Information,” cell-biologist-turned-historian Karl Matlin mentions that while Blobel’s (1980) publication “Intracellular Protein Topogenesis” was only cited “a significant but certainly not unprecedented number” of times (Matlin, 2022, p. 233), this paper was very influential for the field of cell biology. According to Matlin “it is possible that Blobel’s articulation of the concept in specific and concrete terms created a vocabulary for laboratories working in diverse areas, enabling them to focus their work and test specific hypothesis, even if they did not cite Blobel.” (Matlin, 2022, p. 233). Matlin is suggesting that Blobel’s, 1980 paper provided a language framework as well as ideas that allowed workers to push the field forward. In this publication, we use computational methods of assessing influence to demonstrate that Blobel directly influenced the vocabulary of other researchers in a way that is quantitatively sound and goes beyond traditional citation metrics.

We did use Matlin’s insight as a former cell biologist and former colleague of Blobel to inform and narrow our selection criteria to set up an experiment where we examine those who did cite and discuss Blobel’s ideas. We used this specifically to build collections of relevant work to Blobel’s research and collection representative of the larger field of molecular cell biology at the time. This greatly informed our approach, and thus we are able to use a manageable number of sources to examine the field of molecular cell biology during the 1980s and 1990s. In this way, we design an experiment to detect uncited influence of Günter Blobel’s ideas from his 1980 paper, and to try and validate Matlin’s argument. We thus examine how ideas among the larger community that participated in the same investigations of relevant subject matter—yet did not cite Blobel’s paper—either engaged in discussion of Blobel’s concepts anyway or show no evidence of being influenced by him.

## Overview of methods

*Uncited influence* is identifiable using linguistic tools such as *concordance* and *collocation*. *Concordance* refers to specific terms and how they are used in sentences within a text, and *collocation* refers to the co-occurrence of two terms that are used together in a text. In this paper we define a collocation specifically as the co-occurrence of lexical terms where a *keyword*, or relevant term of interest which stands out when compared to a corpus of standard English at a frequency greater than chance, co-occurs with a collocate of some kind (a term being used within five spaces to the right or to the left of that keyword in each collection or subcollection of text).

We rank all keywords in each subcollection by a standard log-likelihood (or the natural logarithm of the likelihood) algorithm considering BOTH frequency of usage as well as unique features of each subcollection to rank keyword prevalence. The formula for log-likelihood is:

$$ {\mathrm{LL}} = 2^{*}\left( {\left( {{\mathrm{a}}^{*}\ln \left( {{\mathrm{a}}/{\mathrm{E}}1} \right)} \right) + \left( {{\mathrm{b}}^{*}\ln \left( {{\mathrm{b}}/{\mathrm{E}}2} \right)} \right)} \right) $$

where

$$ {\mathrm{E}}1 = {\mathrm{C}}^{*}\left( {{\mathrm{a}} + {\mathrm{b}}} \right)/\left( {{\mathrm{C}} + {\mathrm{D}}} \right) $$

and

$$ {\mathrm{E}}2 = {\mathrm{D}}^{*}\left( {{\mathrm{a}} + {\mathrm{b}}} \right)/\left( {{\mathrm{C}} + {\mathrm{D}}} \right) $$

(from Pojanapunya & Todd, 2018).

Each sampled subcollection in is compared to a collection of basic English to determine overall keyword prevalence, or “keyness,” (Scott, 1997) of terms that characterize a text or collection of texts.

When placed in context to the entire collection or subcollection of text, *collocations* can be quantified and thus comparatively measured within a collection or subcollection (Biber, 1993; Baker, 2023; McGee, 2006; Brezina et al., 2015; Gablasova et al., 2017).

Using keywords and collocates, a pattern of text can thus be represented as a *node* in a network within a time series. If a pattern of language is specific to a particular area of work, but that pattern doesn’t exist before or after a certain point, we can theoretically find the *boundaries* of this idea—in particular its *origin*. Thus, a *collocation* can serve as a *fingerprint* of influence, connecting ideas to earlier and later work through *edges* which connect the *nodes* of the network, and where there are *specific boundaries of usage demonstrated by changes in the collocation measurements*. In citation networks a connection between two *nodes* requires a *specific citation* of source material. However, with *collocation networks* as described above mere *patterns of words* can serve as *edges* connecting documents (or *nodes*) where that pattern is present, and *fluctuation* of usage can be characterized by analyzing the patterns of collocation to determine *directionality* of pertinent ideas. The *boundaries* are indicated by the *origin* or *cessation* of these *collocation patterns* across sources or over time, and *locating these boundaries* serves to indicate a source of *initial influence*. This network analogy can serve as a literal representation of these connections, but in this paper we will not use network analysis. It is for heuristic/visualization purposes to represent what we are trying to do here.

Here, we are trying to characterize the language of molecular cell biology in the 1980s and 1990s and identify the *boundary* indicating a pattern of language usage which can be attributed to Blobel regarding his concept of protein topogenesis, though the *language* signifying this concept won’t necessarily involve the term itself. By building a collection of texts for comparison from different time periods and quantifying the collocations, particularly for terms connected to Blobel’s ideas, we can thus test whether such concepts discussed by Blobel and his peers *become relevant to the rest of the molecular cell biology community or not*. We used WordSmith Tools 7.0 software to build keyword lists and perform collocations (Scott, 2016). For more information on materials, methods, collocation and keyword lists, see the corresponding author’s thesis (Jinson, 2024 pp. 1–24, 35).

## Building a collection

Matlin writes that Blobel’s term “protein topogenesis,” or the binding of proteins to the cell membrane and transport to different locations, was an influential idea. However, as stated above this does not necessarily mean that workers are going to be using Blobel’s explicit terms, especially if they aren’t citing the paper. For example, “protein topogenesis” returns very few results when querying Scopus (for those unfamiliar with Scopus, it is a subscription-based database of scientific articles categorized by citations, keywords and concepts)—much smaller than the yet-underwhelming number of papers which cited Blobel’s work. So, “protein topogenesis” is not the keyword we are after to search for nuanced influence. Additionally, the corresponding author of this paper previously demonstrated how the term “topogenic sequences” and even the term “protein topogenesis” Blobel defined in the 1980 paper were never influential terminology picked up and used by many other researchers—not even by those citing his ideas (see Jinson, 2024 pp. 30–31). Therefore, instead of looking for explicit mentions of the topogenesis concept, we looked for terms present within *definition* of his term *rather than the term itself*—such as “cell,” “membrane,” “proteins,” “transport,” and even “binding” or “bound” as well as terms relating to location such as “inner,” “outer,” and “integration.” By locating such *collocates* for prominent keywords, we can find a *fingerprint* for the language of Blobel’s peers who were influenced by his ideas. Following this, we can try to find these same patterns among other literature from the larger field over time to find how the rest of the community (not just those citing Blobel) were talking about these ideas around the time of Blobel’s paper and during the following decades, and if ideas from Blobel and his peers could be traced to having any influence over the language of the larger community as Matlin argues they did.

We sought to identify Blobel’s influence among the publications from molecular cell biology during the 1980s and later. To do this, we needed (A) a *Research collection* that characterizes the larger molecular cell biology community from the time after Blobel’s publication, sampled over time, (B) publications from the same sampling periods as the Research collection *directly citing Blobel*, titled the *Blobel collection*, and (C) a control corpus of some kind sharing the same common knowledge, yet referencing specifically different ideas than those in the *Blobel collection* (not citing Blobel’s, 1980 paper). We thus probe the language of “cells,” “membranes,” “proteins” or other common keywords related to Blobel’s concept of *membrane binding* and *protein transport* used by those talking about Blobel’s work and estimate whether there was some kind of language pattern regarding these concepts originating from the *Blobel collection*, potentially demonstrating directionality of influence. “Cell,” “membrane,” and “protein” were terms that remained consistently in the top keywords (as ranked by log-likelihood) across different time points when sampling the whole collection from 1984 to 2013. We used these as keywords to find collocates for them specifically because, as they remained as consistent entities over time, collocates for them better represent the subject(s) of study to examine the language patterns around them versus other inconsistently used keywords. Focusing on collocates for highly represented keywords that are consistently used over time helps to get at characterizing the ideas presented in these papers and allows us to describe how usage patterns might change over time.

To qualify a sampled collection as representative of the larger field of molecular cell biology beyond those intimately working with Blobel, we needed reliable qualitative input. Matlin provided us with a list of six or seven terms (through personal communication) which—in his experience as a former molecular cell biologist—comprised the areas of work he and his colleagues believed Blobel’s ideas penetrated. We searched Scopus for papers associated with specific keywords to find directly relevant and well-represented keywords to capture the larger field working in molecular cell biology which was intimately tied to the same field as Blobel and his network of citations. We selected sampling periods of five-to-ten-year intervals following the publication of Blobel’s (1980) paper from which we would collect publications for the research collection. We chose five relevant keywords, sampling Scopus publications ranked by number of citations from five different time periods for each of these (Table 1), sampling one-hundred and four different documents over five time periods in total (Table 2). Interestingly, none of the collected papers directly cited Blobel’s, 1980 paper. We referred to this sampling as our *Research collection*. As they did not cite Blobel’s paper yet were pertinent to the key terms Matlin specifically laid out, we thought this a satisfying representation of the field with which to probe Blobel’s influence.

**Table 1:** Our modified list of terms based on Matlin’s qualitative suggestions

Relevant indexed keyword from Scopus database	# of Scopus search results
“protein localization”	208,850
“protein sorting”	11,303
“protein secretion”	77,253
“secretory pathway”	11,226
“secretory protein”	11,869
Total	320,501

We used these terms to construct our Research collection. Each term returned more than 10,000 results—far more than Blobel’s terms such as “protein topogenesis”—which only returned 51 results and “topogenic sequence” which only returned 61 results

We also sampled those citing Blobel’s (1980) paper from the same five time periods to form the *Blobel collection*, comprised of one-hundred and twenty-seven documents. Previously published work helped to identify a control corpus of related but distinct material with which to compare how the language of another group of authors other than Blobel’s network may have influenced or potentially stood as a counterexample influencing the Research collection (Jinson, 2024 pp. 37–38). This consisted of citations of Meyer et al.’s, 1982 paper on protein docking (Meyer et al., 1982), and Perlman & Halvorson’s 1983 paper on signal peptides (Perlman & Halvorson, 1983), therefore we also sampled citations of these two papers from the same time periods as the other two collections and called it the *Meyer & Perlman* or *MP collection*, comprised of one-hundred and nineteen documents. The three collections (and subcollections of the five sampling periods for each collection) are laid out in Table 2. We collected the pdfs of these papers, converted them to text, cleaned them, and analyzed them using WordSmith Tools 7.0 (Scott, 2016).

**Table 2:** The three collections, sampled from specific time periods

Collection	1984–1986	1988–1990	1995–1996	2002–2005	2010–2013
Research	19	19	21	23	22
Blobel	23	12	24	37	31
Meyer & Perlman	34	32	26	17	10

Each cell represents the number of texts we collected and examined for a specific subcollection: for example, the cell corresponding to the “Research” row and “1988–1990” column is a 19-text subcollection of the “Research” collection. The Research collection consists of the most cited papers on Scopus for each time window per keyword listed in Table 1. The “Blobel” collection is comprised of papers citing Blobel’s (1980) article published during these time periods, and “Meyer & Perlman” is comprised of papers from these time periods citing Meyer et al.’s (1982) paper and/or citing Perlman and Halvorsen’s (1983) paper

## Results

**Fig. 1:**
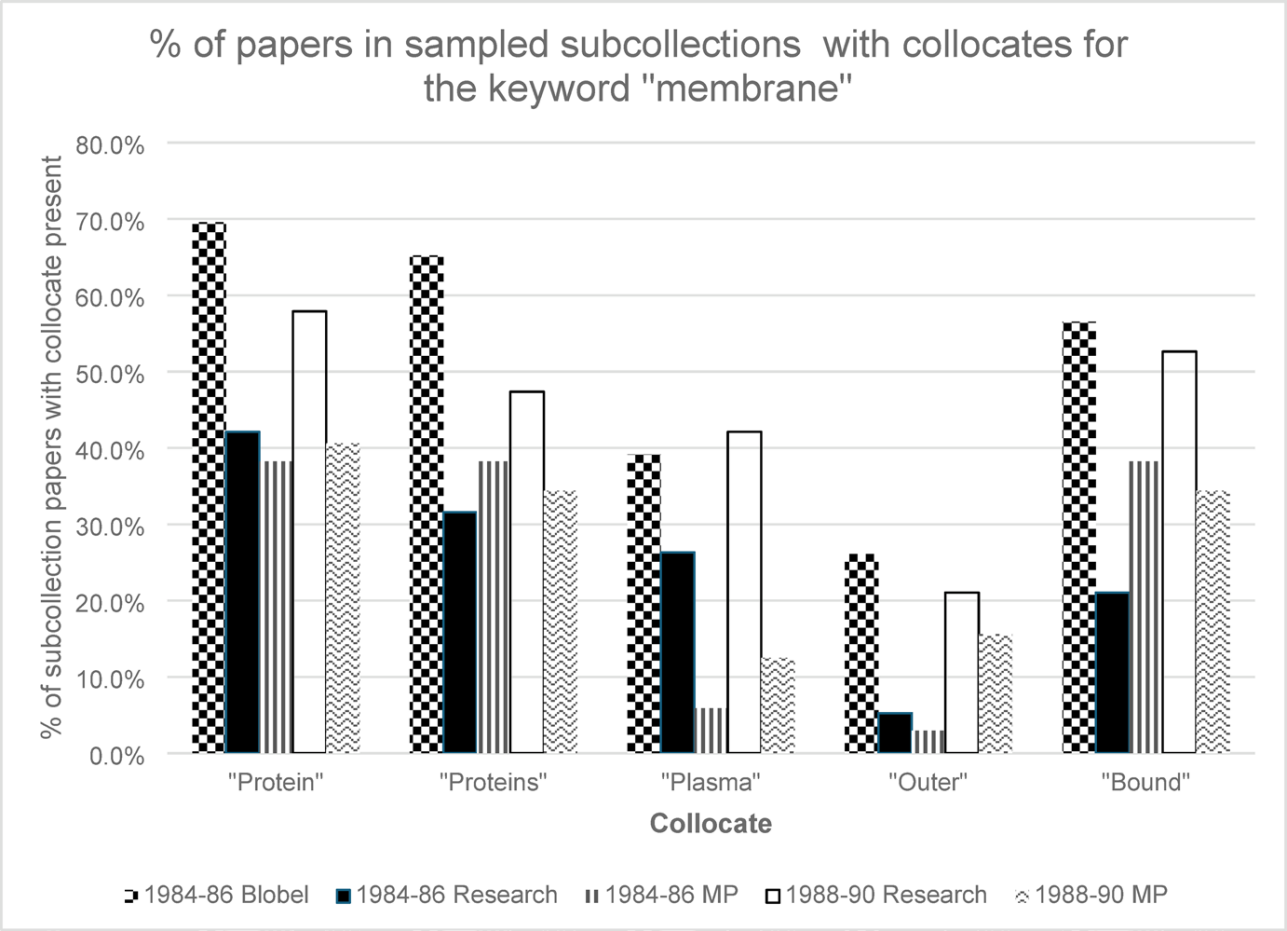
Percent of papers from each subcollection where specific collocates for “membrane” were present for five subcollections. Notice that the threshold (1984–1986 Blobel, checkered) is exceptionally high compared to the other four subcollections here other than 1988-90 (no fill), and this graph demonstrates how the Research subcollection from 1988 to 1990 very closely emulates those citing Blobel’s (1980) paper from 1984 to 1986 (around five years after it was published) with all of the top collocates from the papers where researchers were discussing Blobel’s ideas and discussing his ideas regarding spatial organization of the cell

**Fig. 2:**
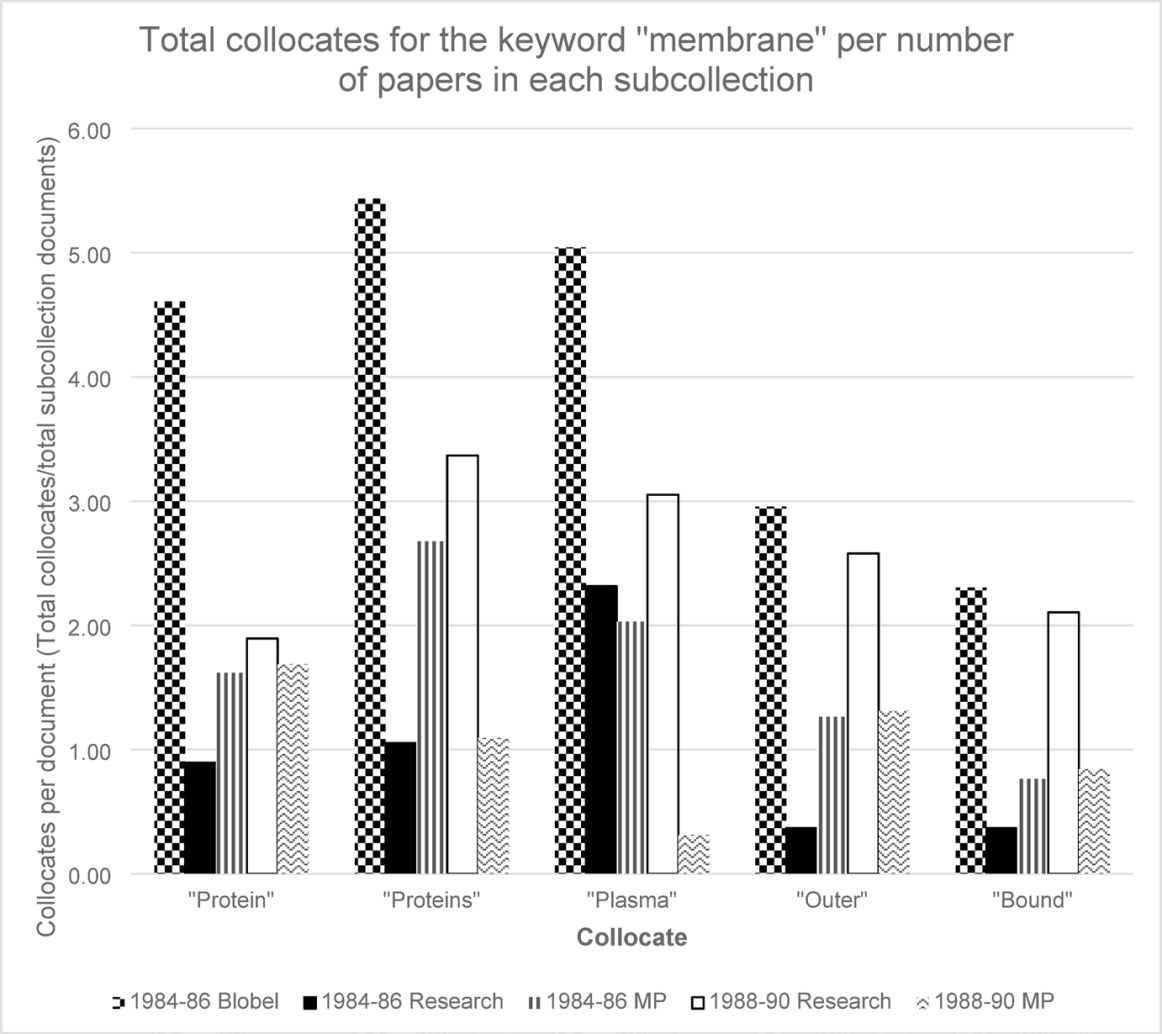
Similar to Fig. 1, but with overall collocate numbers as opposed to Fig. 1, which demonstrated the percent of papers using the collocate in the collection

“Proteins” and “membranes” proved to be the most prominent keywords in all three collections along with the term “cell.” Thus, *collocations* or patterns of terms used together with these specific keywords from each collection over time were measurable, as there was ample representation of these keywords, but distinct *patterns* of collocates for each subcollection when compared to others. Specifically, the concepts of “membrane proteins,” “membrane bound,” “outer membrane,” and “protein binding”—all central to Blobel’s *concept and definition* of “protein topogenesis” even if they didn’t adopt his terms or reference his paper—was a *clear fingerprint of Blobel’s work echoed by those who had actually cited him five years earlier* (Figs. 1 and 2). Notice in these figures the prevalence of the keyword “membrane” appearing with the collocates “protein,” “proteins,” “plasma,” “outer,” and “bound” in the 1988–1990 research subcollection when compared to the 1984–1986 Research subcollection. Of the five subcollections displayed in the figures, the 1984–1986 Blobel subcollection often has the highest usage of the five collocates (as these collocates were chosen to measure because they were the most prevalent collocates in that Blobel subcollection). However, while the 1984–1986 Research subcollection demonstrated very low usage of these same terms, the 1988–1990 Research subcollection is by far the closest to the usage pattern demonstrated by the 1984–1986 Blobel subcollection, which is addressing exactly the things Blobel was talking about in his 1980 paper which Matlin is referring to as influencing the field.

MP is important as an example of another collection related to Blobel and the Research collection’s subject matter but not articulating the same general ideas that Blobel was putting forward. MP’s ideas and the ideas of those citing them were very immediately technical and not as general as those of Blobel’s in providing a way to think about the organization of the cell. In Figs. 1 and 2, the Blobel subcollection from this period clearly uses Blobel’s terminology prominently, while those not citing Blobel (the Research and MP subcollections from this period) used these terms relatively infrequently. Then in 1988–1990, the Research subcollection nearly matches the usage patterns of the collocates (and eclipsing those of other potential influencers measured here such as MP) that those citing Blobel five years previous were using most prominently, despite the astonishing fact that none of these papers in the 1988–1990 Research subcollection cited Blobel’s (1980) paper (only five of the papers cited works with Blobel’s name on the paper, but none of them cited “Intracellular Protein Topogenesis”).

**Fig. 3:**
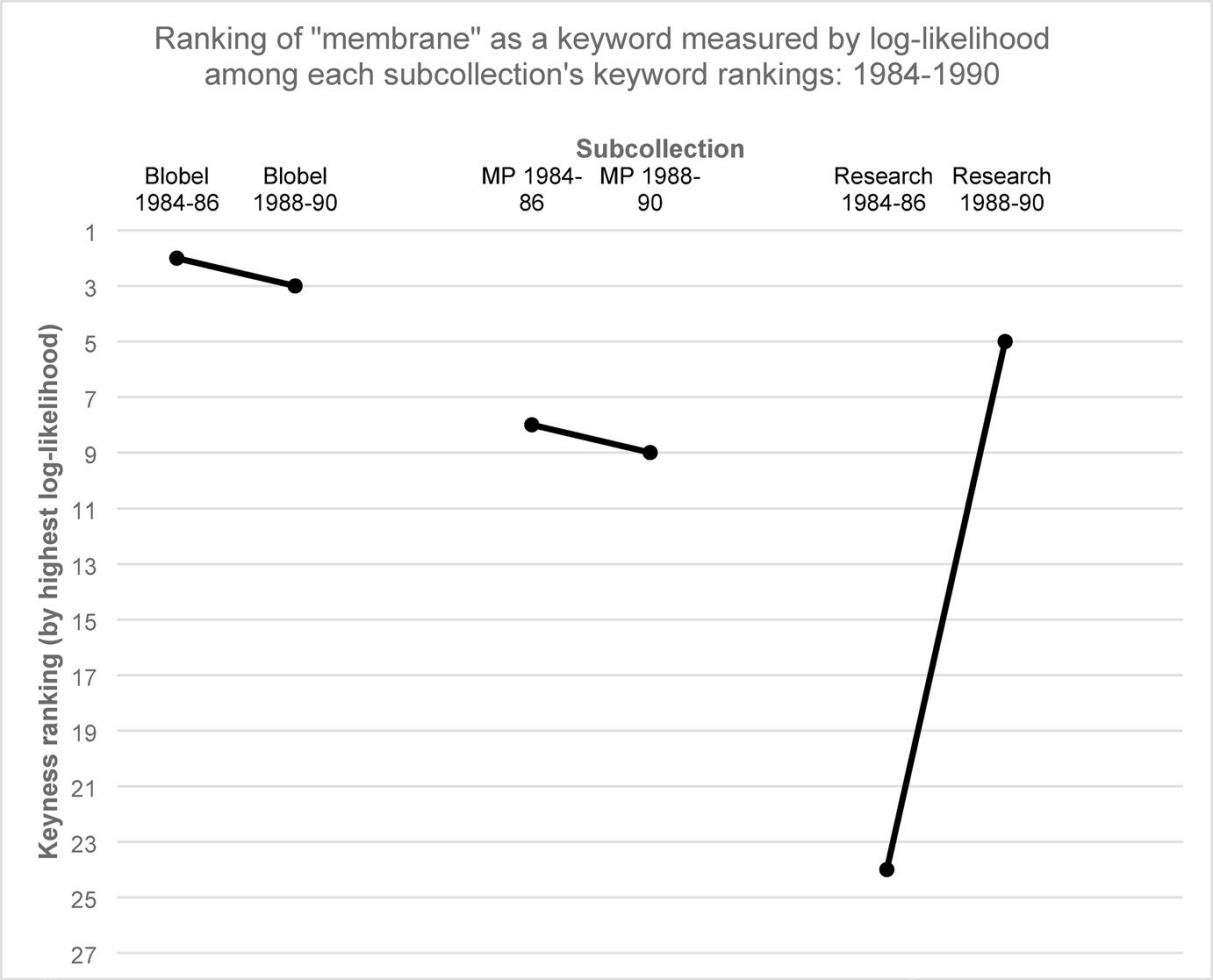
Line graph showing relative trajectories of the keyword “membrane” in all three collections between 1984 and 1990. Notice the “rapid ascent” of the term in the research collection. This surpassed the MP collection rankings, demonstrating further evidence that it was Blobel, not Meyer, Perlman, and Halvorsen, whose influence shaped the patterns of the Research collection in 1988–1990 five years later

In terms of keyness, “membrane” in the Research subcollection for 1988-90 also eclipses that of the MP subcollections from 1984 to 1990 (Fig. 3). In fact, “membrane” as a keyword is ranked fifth overall by log-likelihood—very similar to the ranking of membrane in the Blobel subcollections of 1984–1990. Those citing Blobel five years previously thus not only spoke a collective language echoing Blobel’s ideas, but heralded the changes in collocation for the Research subcollection around five years later. This language is therefore largely not prevalent beyond those discussing Blobel’s ideas in the mid-1980s, but then picked up at a rapid pace five years later by the larger field *without citing Blobel directly*, *as none of the Research collection papers directly cite* Blobel’s (1980) *paper*. This pattern does not exist in the larger field before the late 1980s, does not appear elsewhere at the same rates that those talking about Blobel’s work are discussing it, but is in abundance among the larger survey of literature five years later. This is clearly a fingerprint of Blobel’s influence.

**Fig. 4:**
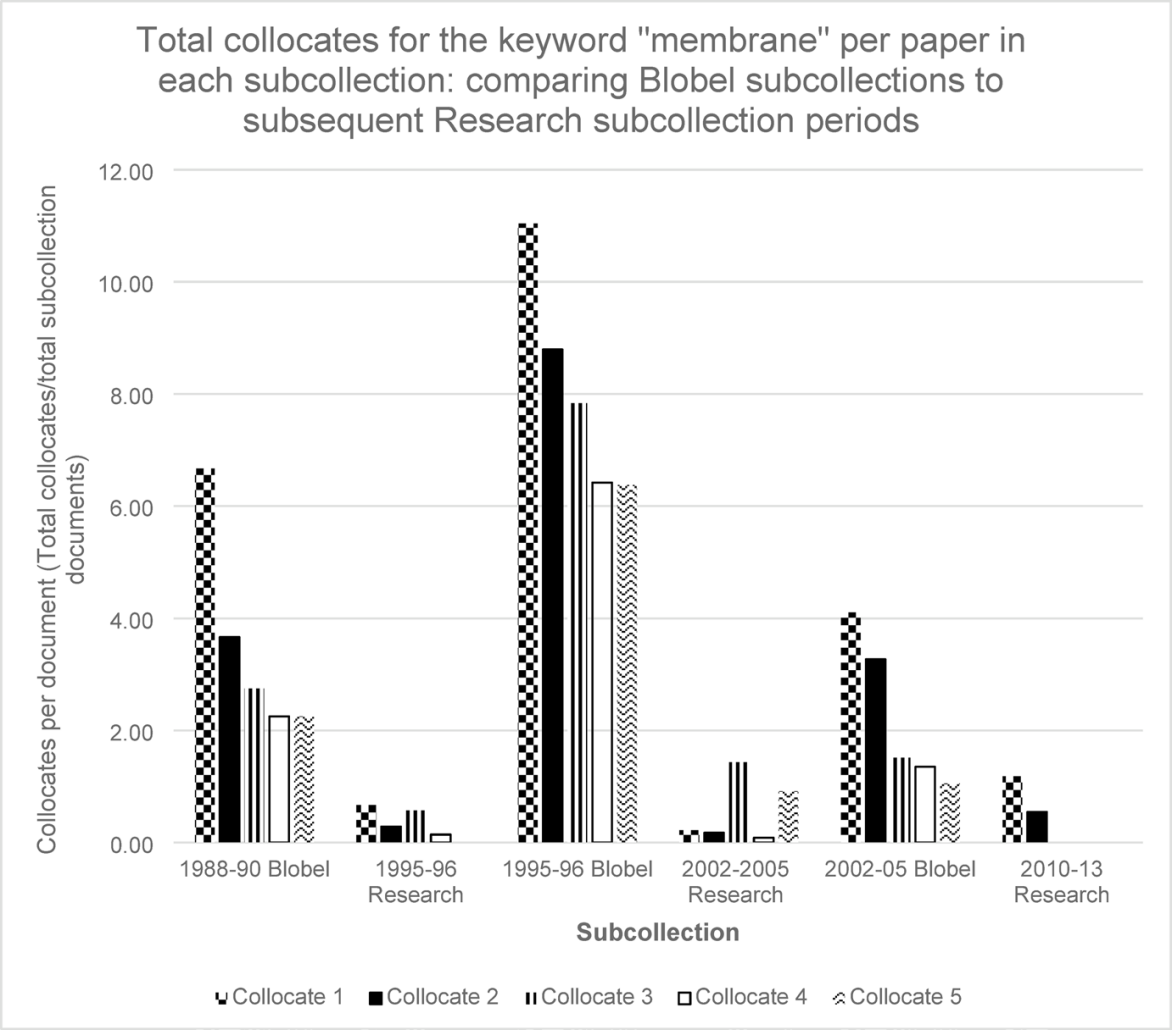
Collocates varied depending on the Blobel corpus in the previous period, but in this figure the previous period’s top five collocates were measured, and then those same collocates were measured in the Research collection from the subsequent time period. In Figs. 1 and 2, we showed how the 1988–1990 subcollection was very similar (in collocate usage patterns of all five top collocates) to the Blobel 1984–1986 subcollection. Here, we see that this similarity between these two subcollections (which we characterize in this paper as an uncited influence that Blobel had on the 1988–1990 subcollection as demonstrated by a similar collocate fingerprint between the two) peaked therein, and the chart above shows how there is a rapid decline in similarity from 1995 on. This shows that they are not talking about the same things. Additionally, the changing number of collocates over time in the Blobel collections indicate they are talking about membranes differently from the 1990s on in this collection as well

However, such uncited influence should not be expected to last forever. In fact, Fig. 4 shows the decline in prevalence of collocates we sampled that were most prevalent in papers published by those still talking about Blobel’s (1980) paper from between 1995 and 2005 compared to the respective Research subcollection from the subsequent sampling periods (over 2002–2013), and the fingerprint becomes weaker and weaker. Considering that the collocates in the Blobel collection changed over time, it is clear that even those citing Blobel are talking about his paper differently at different points of time. Therefore, why would we expect *uncited* influence to extend for decades if not even the *citing* works demonstrate the same patterns over time?

## Conclusion

We show here a relatively simple experiment where the influence of ideas as demonstrated through examining patterns of language is detectable and quantifiable. The influence of an author is not necessarily demonstrated by *what* is being studied but *how* it is being studied and especially how people are talking about it at any given time. We used a qualitative source as a probe to limit the scope of relevant collocations to observe. We next established a pattern of usage indicating that those discussing Blobel’s paper were using the language related to his definitions rather than his explicitly offered terminological keywords such as “protein topogenesis” and “topogenic sequence,” and set up an experiment where we measured and compared collocations to define a boundary of influence between those citing Blobel and not citing Blobel during the 1980s. Then, we used the quantitative measurements to establish directionality and thereby define a gradient of influence indicating an origin point in the mid-to-late-1980s, when Blobel’s language regarding his concepts presented in his 1980 paper penetrated the language of the larger community. These results thus confirm Matlin’s qualitative impressions by showing quantitatively the nature of the influence.

By the mid-1990s, these patterns of collocation wane, even among those citing Blobel. New collocates of “membrane” and “protein” such as “fusion,” “insertion,” “import,” and “topology,” (unpublished data) paired with the absence of previous collocates from earlier periods, as well as changing usage of collocates in Fig. 4 indicate *these keywords are being used in different ways and not demonstrating the same pattern of influence as in* Figs. 1 and 2. For example, in Fig. 4 the *2010–2013 Research collection has zero collocates for three of the top five collocates those citing Blobel discussed in 2002–2005*. The reasons for citing Blobel’s (1980) paper in the 2000s and 2010s are likely specifically changing as well, *as indicated by these changing patterns*. As the life sciences are constantly pursuing new questions, it is reasonable to conclude that *the semantic influence of every writer has a limit*, and many of these limitations can likely be detected in language patterns as we have shown in this case.

## Data Availability

Many journal articles used for this study were available through open access, but some articles and use of the Scopus database—as well as software such as WordSmith Tools—were available through institutional subscription only.
